# Too many digits: the presentation of numerical data

**DOI:** 10.1136/archdischild-2014-307149

**Published:** 2015-04-15

**Authors:** T J Cole

**Keywords:** Statistics, Measurement

Emperor Joseph II: My dear young man, don't take it too hard. Your work is ingenious. It's quality work. And there are simply too many notes, that's all. Just cut a few and it will be perfect.

Mozart: Which few did you have in mind, Majesty?

Emperor Joseph II: Well, there it is.

Quotation from the film *Amadeus* (1984)

As a statistical reviewer for *Archives* and *BMJ* I am interested in the presentation of numerical data.^1^ It concerns me that numbers are often reported to excessive precision, because too many digits can swamp the reader, overcomplicate the story and obscure the message.

A number's precision relates to its decimal places or significant figures (or as preferred here, significant *digits*). The number of decimal places is the number of digits to the right of the decimal point, while the number of significant digits is the number of all digits ignoring the decimal point, and ignoring all leading zeros and some trailing zeros (for a fuller definition see ‘significant figures’ on Wikipedia).

Ideally data should be rounded appropriately, not too much and not too little (one might call it Goldilocks rounding).^2^ The European Association of Science Editors guidelines include the useful rule of thumb: “numbers should be given in (sic) 2–3 effective digits”.^3^

Take as an example the odds ratio (OR) of 22.68 (95% CI 7.51 to 73.67) comparing beta mimetics with placebo for side effects requiring a change of medication.^4^ Its two decimal places and four significant digits are excessive when the effect size and confidence interval (CI) are so large. Reporting it rounded to two significant digits, as 23 (7.5 to 74), or even as 23 (8 to 70), with one significant digit for the CI, would be simpler and clearer.

There are several published recommendations (or *reporting rules*) about rounding numbers, some of which relate to decimal places (eg, the Cochrane Style Guide^5^ or APA Style^6^ to round to two decimal places), some to significant digits (eg, the European Association of Science Editors guideline above^3^) and some to a combination of the two (eg, setting the number of decimal places to ensure two significant digits for the standard deviation (SD)^7^). However, the message here is that rules of the first type, specifying the number of decimal places and ignoring the number of significant digits, are inherently unsatisfactory, as the following examples show.

Birth weight is usually reported in units of grams, for example, “birth weight … resulting from blastocyst transfer was significantly greater than … resulting from Day 3 transfer (3465.31±51.36 g vs 3319.82±10.04 g respectively, p=0.009)”.^8^ However it is also reported in kilograms: “The mean birth weight of babies was 3.05±0.57 (95% CI 2.95 to 3.15) kg”.^9^ In both articles birth weight is reported to two decimal places, but due to the different units they correspond to six and three significant digits, respectively. The first is clearly excessive while the second is about right, giving the SD to two significant digits.^7^ By analogy, birth weight in grams ought to be rounded to the nearest 10 g.

A second example is the Cochrane Style Guide, which requires risk ratios to be reported to two decimal places.^5^ This is clearly unsatisfactory for ratios that are very large (see the example above) or very small, for example a hazard ratio (HR) of 0.03 (95% CI 0.01 to 0.05) for the updating of systematic review citations in Clinical Evidence versus Dynamed.^10^ If the direction of the HR were reversed its true value could be anywhere between 29 and 40 due to the extreme rounding.

As a third example, p values, it has been suggested, should be rounded to one or two decimal places.^2^ For p values above the conventional 0.05 cut-off there is little justification for quoting more than one decimal place, while for significant results three or even four decimals may be necessary. The better rule is to report rounded up to one significant digit, which works across the spectrum of values.^1^

Thus a decimal places rule that ignores significant digits does not work. But equally, and perhaps surprisingly, a significant digits rule that ignores decimal places does not always work either. Reporting risk ratios to three significant digits for example leads to the largest ratio below 1 being reported as 0.999 and the smallest above 1 as 1.01, with three and two decimal places, respectively. This is clearly unsatisfactory as they differ in precision by a factor of ten. In this instance a combination of significant digits and decimal places, the rule of four,^11^ works best: round the risk ratio to two significant digits if the leading non-zero digit is four or more, otherwise round to three.

The rule of four gives three decimal places for risk ratios from 0.040 to 0.399, two from 0.40 to 3.99 and one from 4.0 to 39.9.^11^ Applying it to the example of 22.68 above gives 22.7 (95% CI 7.5 to 74). Alternatively one can apply the rule with one less significant digit, giving 23 with CI 8 to 70.^11^

Another example is the reporting of test statistics such as t or F. Specifying one decimal place would permit say t=30.1, where 30 is clearly sufficient as it is so highly significant. Conversely specifying two significant digits would permit t=−0.13, where again the extra precision is irrelevant as it is far from significant. A suitable rule specifies up to one decimal place and up to two significant digits.

When comparing group means or percentages in tables, rounding should not blur the differences between them. This is the basis for the Hopkins two digits rule,^7^ whereby the mean has enough decimal places to ensure two significant digits for the SD. An analogous rule for percentages might be to use enough decimal places to ensure two significant digits for the range of values across groups, eg, if the range is 10% or more use whole numbers, if less than 1% use two decimal places, and otherwise one. In practice percentages are usually given along with their corresponding frequencies, so precision is less critical as the exact values can be calculated.

Recognising the fallibility of decimal places rules means that tables ought not to be restricted to columns of numbers with fixed decimal places, and this adds flexibility when deciding how many decimals to use. For example measures of variability, eg, standard errors (SE)s or CIs, need not be as precise as the effect size, particularly if the CI is wide. A useful trick when formatting table columns is to align the numbers by decimal point, which highlights differences in the number of decimal places. This is particularly useful in columns of risk ratios or p values—see the examples in the table.

It is important that any intermediate calculations are carried out to full precision, and that rounding is done only at the reporting stage. This raises the question as to whether the rounded results need extra precision in case they are later included in meta-analyses. But to my mind this confuses two distinct aims—to present the results as accessibly as possible, and to report the results as raw data for meta-analysis. If the two aims are mutually exclusive then the first has to take priority. But in practice they may not be mutually exclusive—where effect sizes are rounded to two or three significant digits the rounding error variance is likely to be small compared with the inter-study variance, making the rounding imprecision relatively unimportant.

Table 1 gives an evidence-based set of recommendations for rounding the types of summary statistic that arise commonly in medical scientific writing. The general principle is to use two or three significant digits for effect sizes, and one or two significant digits for measures of variability.^3^ ^12^ However, optimal precision, like beauty, is in the eye of the beholder, and they should be recognised as recommendations not requirements. Their main purpose is to address the pervasive problem of reporting too many digits.

**Table 1:** Rounding rules for summary statistics

Summary statistic	Reporting	Examples (where useful)
Mean	Use enough decimal places to give either the SD to two significant digits,^7^ or the SE to one significant digit	3320 g
		3.32 kg
Percentage	Integers, or one decimal place for values under 10%. Values over 90% may need one decimal place if their complement is informative. Use two or more decimal places only if the range of values is less than 0.1%	0.1%
		5.3%
		27%
		89%
		99.6%
Mean difference	Use enough decimal places to give the SE to one or two significant digits. For a standardised mean difference use one or two decimal places	
Regression coefficient	As with the mean difference.	
Correlation coefficient	One or two decimal places, or more when very close to ±1	0.03
		−0.7
		0.89
		0.999
Risk ratio	Round to two significant digits if the leading non-zero digit is four or more, otherwise round to three (the rule of four^11^). Alternatively use one/two significant digits rather than two/three. For ORs very close to 1 (eg, in logistic regression with a continuous variable) use three decimal places or else report the log OR×100 as the percentage odds to one decimal place^13^	0.0321
		0.062
		0.76
		1.05
		4.2
		11.3
		55
		1.042
		4.1%
SD	One or two significant digits^7^	570 g
		0.57 kg
		9 mm Hg
		2.5 mL
SE	One or two significant digits	
CI	Use the same rule as for the corresponding effect size (be it mean, percentage, mean difference, regression coefficient, correlation coefficient or risk ratio), perhaps with one less significant digit	
Test statistics: t, F, χ^2^, etc	Up to one decimal place and up to two significant digits	
		t=−1.3
		F=11
		χ^s^=4.1
p value	Round up to one significant digit, within the limits shown in the examples. The lower limit may be smaller than 0.001, but never 0.000. For genome-wide association studies use the power of 10 format	>0.9
		0.4
		0.1
		0.08
		0.05
		0.003
		<0.001
		6.10^−9^

Fortunately for us, Emperor Joseph did not tell Mozart which notes he should cut, but here researchers have a clear steer as to which digits they can ditch.
